# Real-world efficacy and safety of PD-1 inhibitors in patients with advanced esophageal squamous cell carcinoma: a single-center retrospective analysis

**DOI:** 10.3389/fonc.2025.1658010

**Published:** 2025-10-29

**Authors:** Xin Wang, Lili Jia, Yijian Zhao, Qinqin Wang, Yang Zhou, Yu Wang, Xialu Zhang, Miaoxu Zhai, Yinghong Ren

**Affiliations:** ^1^ Department of Oncology, Shangluo Central Hospital, Shangluo, Shaanxi, China; ^2^ Department of Pharmacology, Shangluo Central Hospital, Shangluo, Shaanxi, China

**Keywords:** esophageal squamous cell carcinoma, PD-1 inhibitors, real-world evidence, clinical outcomes, prognostic factors

## Abstract

**Background:**

Esophageal squamous cell carcinoma (ESCC) is associated with high mortality and limited treatment options. While PD-1/PD-L1-targeted immunotherapy has shown promise, clinical trial results may not fully represent real-world outcomes.

**Methods:**

This retrospective study at Shangluo Central Hospital analyzed 116 patients with ESCC treated with PD-1 inhibitors from April 2021 to December 2023. Data were collected from electronic records to assess clinical outcomes, including overall survival (OS), progression-free survival (PFS), objective response rate (ORR), and disease control rate (DCR), as well as factors associated with treatment response and toxicity.

**Results:**

The cohort had a median age of 66 years, with 86.2% male patients and 71.5% smokers. The majority of patients had advanced disease (stage III: 49.1%, stage IV: 29.3%). The ORR was 40.5%, with 1.7% achieving complete response and 38.8% partial response. The DCR was 81%. The median PFS was 13.6 months, and the median OS was not reached. Better outcomes were associated with age <70 years, ECOG performance status 0/1, fewer than two metastatic organs, and first-line treatment. Treatment-related adverse events (TRAEs) were reported in 10 out of 116 patients (8.6%). Grade ≥3 TRAEs occurred in 4 patients (3.4%), including cutaneous capillary hemangioma (n=3, 2.6%) and dyspnea (n=1, 0.9%). No treatment-related deaths were reported.

**Conclusions:**

In this real-world cohort, PD-1 inhibitors demonstrated notable efficacy and manageable toxicity for ESCC. Younger patients, those with better performance status, and fewer metastases achieved better outcomes. Larger, multi-center studies with biomarker analysis are warranted to validate these findings.

## Introduction

Esophageal cancer is a devastating malignancy characterized by high mortality rates and limited treatment options, resulting in a poor prognosis for affected patients (1, 2). Esophageal squamous cell carcinoma (ESCC) is the predominant histological subtype in many regions, particularly in East Asia (1). Despite advancements in surgical techniques and multimodal therapies, the overall survival (OS) of patients with advanced or metastatic ESCC remains unsatisfactory when treated with conventional chemotherapy alone (typically platinum-based doublets), with median OS often less than a year (1, 2). Current international guidelines, such as those from the National Comprehensive Cancer Network (NCCN) and the European Society for Medical Oncology (ESMO), recommend immunotherapy targeting the programmed cell death protein 1 (PD-1) or its ligand (PD-L1) in combination with chemotherapy as a standard first-line treatment option for advanced or metastatic ESCC, and as monotherapy in subsequent lines, based on results from landmark clinical trials (3, 4).

The advent of immunotherapy has revolutionized cancer treatment by offering a novel approach that harnesses the patient’s immune system to combat cancer cells. Among immunotherapy strategies, targeting the PD-1/PD-L1 pathway is particularly promising. PD-1 and PD-L1 suppress T-cell activation, helping cancer cells evade the immune system (5, 6). Several monoclonal antibodies against this pathway have demonstrated efficacy in various malignancies, including esophageal cancer, thereby highlighting the potential of this therapeutic strategy (7, 8). Landmark phase 3 trials such as KEYNOTE-590 (pembrolizumab plus chemotherapy), CheckMate-648 (nivolumab plus chemotherapy or nivolumab plus ipilimumab), and RATIONALE-306 (tislelizumab plus chemotherapy) have established the superiority of PD-1 inhibitor-based combinations over chemotherapy alone in the first-line treatment of advanced ESCC, leading to their widespread adoption in clinical practice (9–11).

While clinical trials provide crucial insights into PD-1 inhibitor efficacy and safety, they often don’t capture the complexities of real-world practice. Factors like diverse patient populations, varied adherence, off-protocol combinations, and comorbidities can significantly impact outcomes, yet are often controlled or unrepresented in trials (12). Therefore, real-world data is essential for understanding immunotherapy’s true effectiveness and safety in diverse patients, offering a more comprehensive evaluation that bridges the gap between trial results and daily clinical decisions (13, 14).

In this retrospective analysis, we aim to evaluate the efficacy and safety profile of PD-1 targeted immunotherapy in ESCC patients within a real-world clinical setting at our center. By analyzing a cohort of patients treated in routine clinical practice, we seek to provide a comprehensive evaluation of PD-1 inhibitors in esophageal cancer and to further inform the clinical application of immunotherapy in this disease context.

## Materials and methods

### Patients

#### Study population and data source

We conducted a retrospective analysis of patients diagnosed with esophageal cancer (histologically confirmed ESCC) treated at Shangluo Central Hospital from April 2021 to December 2023. Data were extracted from the hospital’s electronic medical record (EMR) system, which includes detailed patient information on demographics, clinical characteristics (including AJCC 8th edition staging), treatment history (including specific PD-1 inhibitor used, start and end dates, and any concomitant therapies such as chemotherapy regimens or anti-angiogenic agents), and follow-up (including dates of progression and death, or last follow-up). The study was conducted in accordance with the ethical standards of the institutional review committee and the 1964 Helsinki Declaration, as well as its later amendments or comparable ethical guidelines. Due to the retrospective design of the study and the use of de-identified patient data, the institutional review board waived the requirement for informed consent.

### Inclusion criteria

Eligible patients met the following criteria: (1) a confirmed diagnosis of ESCC via histopathological examination; (2) locally advanced, recurrent, or metastatic disease; (3) received at least one cycle of PD-1 inhibitor-based immunotherapy (monotherapy or combination therapy); (4) available baseline imaging and at least one follow-up imaging assessment for response evaluation, or documented progression/death.

### Exclusion criteria

Patients were excluded if they had: (1) absolute contraindications to immunotherapy, including active systemic infections, uncontrolled autoimmune diseases, ongoing treatment with high-dose corticosteroids (>10 mg/day prednisone equivalent) or other immunosuppressive agents, or a history of severe hypersensitivity reactions to immunotherapy agents; (2) non-squamous cell carcinoma histological subtypes (e.g., adenocarcinoma, neuroendocrine carcinoma, adenosquamous carcinoma, neurolemmoma as per original exclusion); (3) active hepatitis or interstitial pneumonia; (4) incomplete essential data for efficacy or safety analysis.

**Figure 1:**
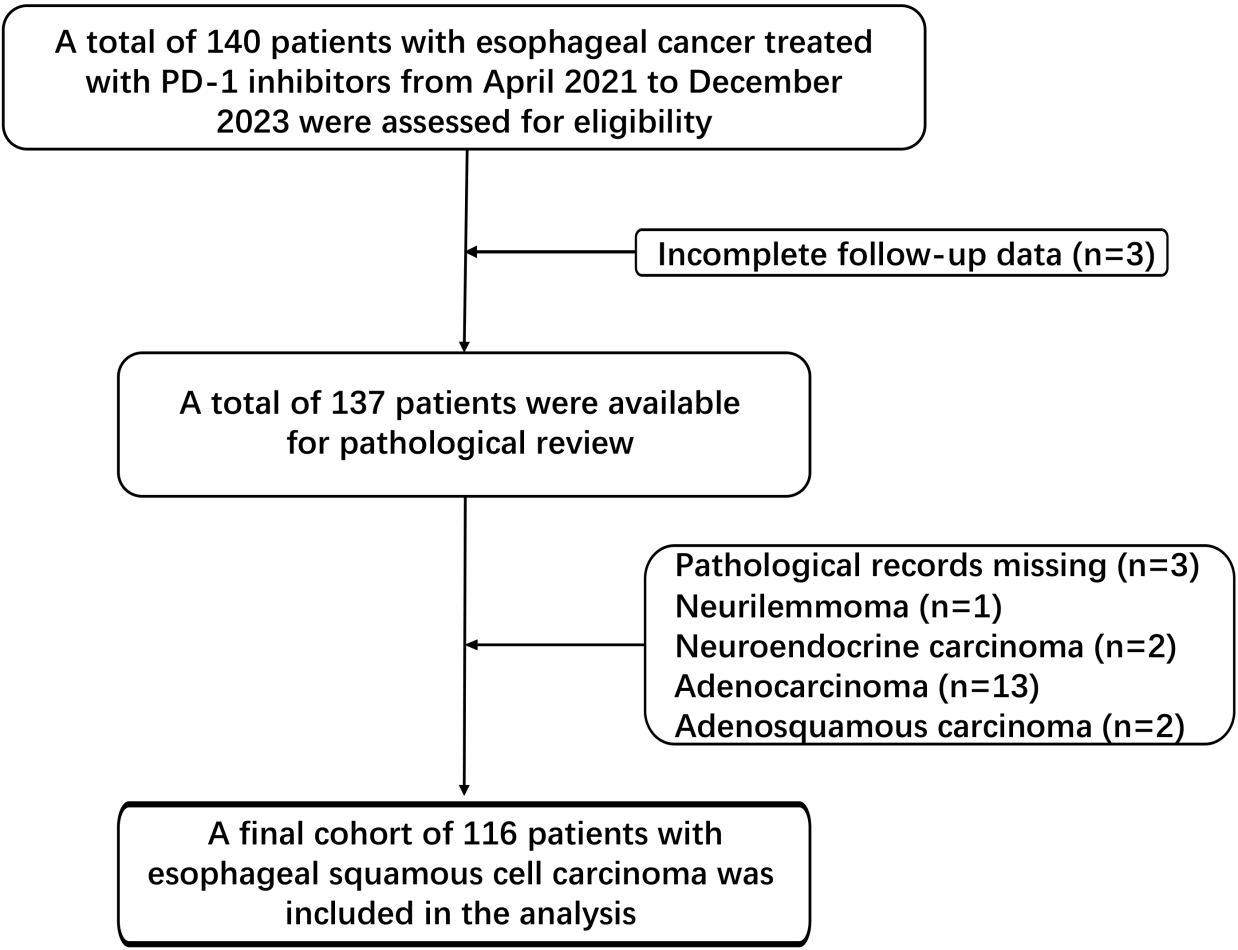
Flow diagram of the study.

**Figure 1** outlines the patient selection process. Of the initial 140 esophageal cancer patients treated with PD-1 inhibitors, 3 were excluded due to incomplete follow-up data, and 21 were excluded due to lacking ESCC pathology or the presence of tumor types. The final cohort consisted of 116 patients.

### Data collection

Data collected included patient demographics (age, gender), clinical characteristics (smoking history, Eastern Cooperative Oncology Group (ECOG) performance status (PS) score, AJCC 8th edition stage, primary tumor location, sites of metastasis including lymph nodes, liver, lung, bone), chronic comorbidities, treatment details (specific PD-1 inhibitor, line of therapy, combination regimen), and pretreatment peripheral blood tests including absolute neutrophil count (ANC), absolute lymphocyte count (ALC), hemoglobin, platelet, sodium, albumin, lactate dehydrogenase (LDH), C-reactive protein (CRP). Notably, the included patients with Stage I/II ESCC were all post-surgical recurrence cases who did not undergo local treatment (surgery/radiotherapy) due to patient-specific factors, including personal preference and the presence of surgical contraindications. Calculations of the neutrophil-to-lymphocyte ratio (NLR), platelet-to-lymphocyte ratio (PLR), and prognostic nutrition index (PNI) were performed as follows: NLR was calculated by dividing the ANC by the ALC, PLR was calculated by dividing the absolute platelet count by the ALC, and PNI was calculated using the formula: 10 × serum albumin (g/dL) + 0.005 × total lymphocyte count (/mm³).

### Efficacy assessment

Tumor response was assessed by imaging (typically computed tomography [CT] or magnetic resonance imaging [MRI]) every 6–8 weeks from the start of immunotherapy, or as clinically indicated, using the Response Evaluation Criteria in Solid Tumors (RECIST) version 1.1 guidelines to categorize patients into complete response (CR), partial response (PR), stable disease (SD), and progressive disease (PD). Objective response rate (ORR) was defined as the proportion of patients achieving CR or PR. Disease control rate (DCR) was defined as the proportion of patients achieving CR, PR, or SD. Progression-free survival (PFS) was defined as the time from the first dose of immunotherapy to the date of documented tumor progression (per RECIST 1.1) or death from any cause, whichever occurred first. Overall survival (OS) was measured from the first dose of immunotherapy to the date of death from any cause. Patients alive without progression or death at the last follow-up were censored for PFS and OS analyses, respectively. The last follow-up was conducted in December 2023.

### Safety assessment

The safety of immunotherapy was assessed by monitoring adverse events (AEs) and serious adverse events (SAEs) throughout the treatment period. AEs were graded according to the Common Terminology Criteria for Adverse Events (CTCAE) version 5.0. Treatment-related adverse events (TRAEs) were defined as AEs considered by the treating physician to be at least possibly related to the PD-1 inhibitor or concomitant therapy. AE data were obtained through retrospective review of EMRs, with documentation made by treating physicians during follow-up visits based on patients’ self-reports and clinical examinations. Reactive cutaneous capillary endothelial proliferation (RCCEP) was diagnosed according to characteristic clinical features without routine pathological confirmation.

### Statistical analysis

Statistical analysis was performed using SPSS 20.0 software (IBM Corporation). Categorical data were expressed as frequencies and percentages. Comparisons between categorical variables were performed using the chi-square test or Fisher’s exact test, as appropriate. Continuous variables were summarized as median (range) or mean (standard deviation) and compared using appropriate parametric or non-parametric tests. Kaplan-Meier survival analysis was used to estimate median PFS and OS and to obtain the 95% confidence interval (CI) values. The log-rank test was used to determine differences in survival between groups in univariate analysis. Variables with a P-value < 0.10 in univariate analysis were considered for inclusion in the multivariate Cox proportional hazards model to identify independent prognostic factors for PFS and OS. Hazard ratios (HRs) with 95% CIs were calculated. A two-sided P-value < 0.05 was considered statistically significant for all analyses.

## Results

### Patient baseline characteristics

**Table 1** summarizes the baseline characteristics of the 116 patients (median age 66 years, 86.2% male). Most were current or former smokers (71.6%) and had moderately differentiated tumors (68.1%). A majority of patients had advanced stage disease (Stage III/IV, 78.4%), T3 tumors (62.1%) and lymph node involvement (N1-3, 69%). Distant metastasis was present in 34.5%. Most had ECOG PS 1 (92.2%). Immunotherapy was primarily administered as first-line treatment (60.3%). Camrelizumab was the most commonly used PD-1 inhibitor (69.8%), predominantly administered in combination with chemotherapy (64.7%), most often taxane-based regimens with or without platinum. Common metastatic sites included lung, liver, bone, and distant lymph nodes. Comorbidities were seen in 40.5%, mainly hypertension. Regarding baseline markers, 16.4% had BMI <18.5, 41.4% had NLR ≥3.62, 33.6% had PLR ≥191.8, and 52.6% had PNI ≥45.05.

**Table 1:** Baseline patient characteristics.

Characteristics	n	%
Age (years)		
range	46-82	
median	66	
<70	76	65.6
≥70	40	34.4
Sex		
male	100	86.2
female	16	13.8
Smoking		
yes	33	71.6
no	83	28.4
Histological grade		
well-differentiated	5	4.3
moderately differentiated	79	68.1
poorly differentiated	8	6.9
unknown	24	20.7
Stage (AJCC-8)		
I	2	1.7
II	22	19.0
III	57	49.1
IV	34	29.3
unknown	1	0.9
T stage		
T0	1	0.9
T1	4	3.4
T2	16	13.8
T3	72	62.1
T4	6	5.2
Tx	17	14.7
N stage		
N0	29	25.0
N1	53	45.7
N2	25	21.6
N3	2	1.7
Nx	7	6.0
M stage		
M0	76	65.5
M1	40	34.5
ECOG PS		
0	2	1.7
1	107	92.2
2	7	6.0
Number of metastatic organs		
0	84	72.4
1	23	19.8
2	7	6
3	2	1.7
PD-1 inhibitor		
camrelizumab	81	69.8
sintilimab	18	15.5
tislelizumab	17	14.7
Therapy regimen		
PD-1 inhibitor monotherapy	15	12.9
Combined with chemotherapy		
taxanes (albumin-bound paclitaxel/docetaxel/paclitaxel) ± platinum	75	64.7
fluorouracil (5-fluorouracil/S-1) + platinum	8	6.9
fluorouracil (capecitabine/S-1)	7	6.0
Combined with anti-angiogenic therapy		
anlotinib	9	7.8
anlotinib + albumin-bound paclitaxel + platinum/S-1	2	1.7
Metastatic site		
lung	19	16.4
liver	12	10.3
bone	10	8.6
distant lymph node	14	12.1
other (stoma/pericardium)	2	1.7
Comorbidities		
diabetes mellitus	4	3.4
hypertension	21	18.1
other	22	19
Line of immunotherapy		
1	70	60.3
2	35	30.2
≥3	11	9.5
BMI (mg/m^2^)		
<18.5	19	16.4
18.5–23.9	80	69
24.0-27.9	14	12.1
≥28	3	2.6
Neutrophil-to-lymphocyte ratio		
<3.62	68	58.6
≥3.62	48	41.4
Platelet-lymphocyte ratio		
<191.8	77	66.4
≥191.8	39	33.6
Prognostic nutrition index		
<45.05	55	47.4
≥45.05	61	52.6

AJCC, American Joint Committee on Cancer; BMI, Body mass index; ECOG PS, Eastern Cooperative Oncology Group Performance Status.

### Efficacy and survival outcomes

Of 116 patients, 1.7% (2/116) achieved CR, 38.8% (45/116) achieved PR, and 40.5% (47/116) had SD, resulting in ORR of 40.5% and DCR of 81%. Nine patients (7.8%) had PD, and 13 (11.2%) were not evaluable.

**Figure 2:**
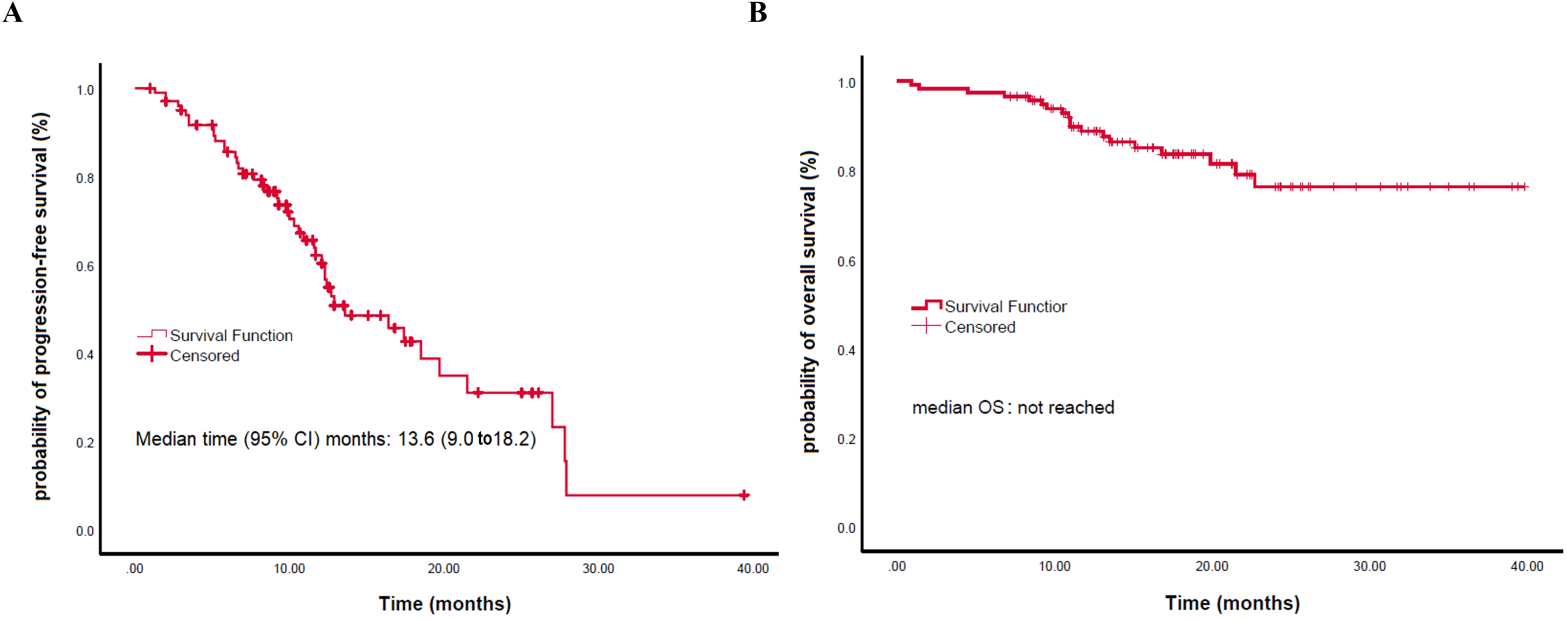
Kaplan-Meier analysis of survival outcomes. **(A)** Progression-free survival (PFS). **(B)** Overall survival (OS).

The median follow-up was 32.4 months (95% CI, 25.4–39.4 months). The median PFS was 13.6 months (95% CI, 9.0–18.2 months) (**Figure 2A**).

Kaplan-Meier analysis showed no significant differences in median PFS or OS among patients treated with different PD-1 inhibitors (camrelizumab: 12.7 months vs. sintilimab: 27.9 months vs. tislelizumab: 10.3 months for PFS, P = 0.547; all not reached [NR] for OS, P = 0.354) (**Supplementary Figure 1**). Similarly, no significant differences were found between immunotherapy monotherapy and combination therapy in median PFS (NR vs. 13.6 months, P = 0.547) or OS (both NR, P = 0.324); the 12- and 24-month PFS rates were 60.4% (95% CI, 48.9–71.9) and 31.0% (16.1–45.9), and the corresponding OS rates were 88.7% (82.7–94.6) and 76.3% (65.9–86.7), respectively (**Supplementary Figure 2**).

**Figure 3:**
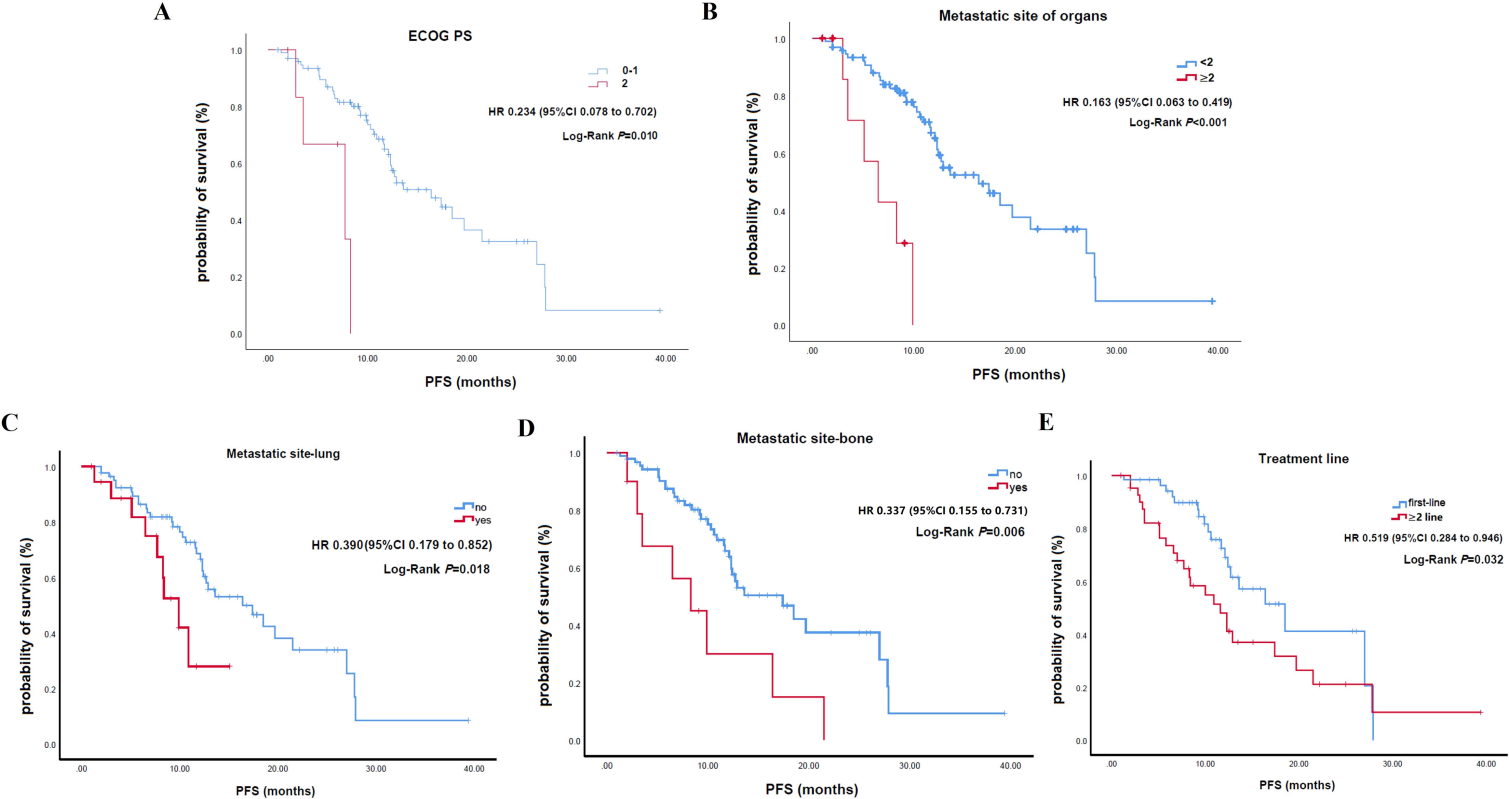
Kaplan-Meier curves for progression-free survival stratified by clinical characteristics. **(A)** ECOG. **(B)** Number of metastatic organs. **(C)** Presence of lung metastases. **(D)** Presence of bone metastases. **(E)** Line of therapy.

In the univariate analysis for PFS, ECOG PS of 0-1 (HR: 0.234; P = 0.010), fewer than 2 metastatic organs (HR: 0.163; P<0.001), absence of lung metastases (HR: 0.390; P = 0.018), absence of bone metastases (HR: 0.337; P = 0.006), and first-line treatment (HR: 0.519; P = 0.032) were significantly associated with better PFS (**Figure 3**; **Table 2**). While other factors like age, sex, smoking, comorbidities, M0 status, liver metastases, distant lymph nodes, and concurrent chemotherapy showed no significant impact, a trend for better PFS was observed in younger patients. In the multivariable analysis, fewer than 2 metastatic organs (HR: 0.146; P = 0.049) and first-line treatment (HR: 0.472; P = 0.028) remained significant independent predictors of superior PFS. Better ECOG PS showed a trend but lost statistical significance in this model (**Table 2**).

**Table 2:** Univariate and multivariate analysis of PFS in the immunotherapy plus chemotherapy subgroup.

Variables	Category	Univariate analysis			*P*	Multivariate analysis			*P*
		HR	95% CI			HR	95% CI		
Age	<70 vs. ≥ 70	0.794	0.406	1.553	0.501				
Sex	Female vs. male	1.414	0.694	2.879	0.340				
Smoking	No vs. yes	1.492	0.689	3.232	0.311				
ECOG PS	0/1 vs. 2	0.234	0.078	0.702	0.010	0.428	0.127	1.448	0.172
Comorbidities	No vs. yes	0.998	0.739	1.347	0.987				
M stage	M0 vs. M1	0.641	0.349	1.177	0.152				
Number of metastatic organs	<2 vs. ≥2	0.163	0.063	0.419	<0.001	0.146	0.021	0.995	0.049
Lung metastasis	No vs. yes	0.390	0.179	0.852	0.018	0.825	0.27	2.521	0.736
Liver metastasis	No vs. yes	0.737	0.308	1.764	0.494				
Bone metastasis	No vs. yes	0.337	0.155	0.731	0.006	1.015	0.271	3.799	0.982
Distant lymph node metastasis	No vs. yes	0.993	0.434	2.272	0.987				
Current regimen	With vs. without chemotherapy	1.233	0.594	2.558	0.575				
Treatment line	1st line vs. ≥ 2nd line	0.519	0.284	0.946	0.032	0.472	0.242	0.921	0.028

ECOG PS, Eastern Cooperative Oncology Group Performance Status.

**Figure 4:**
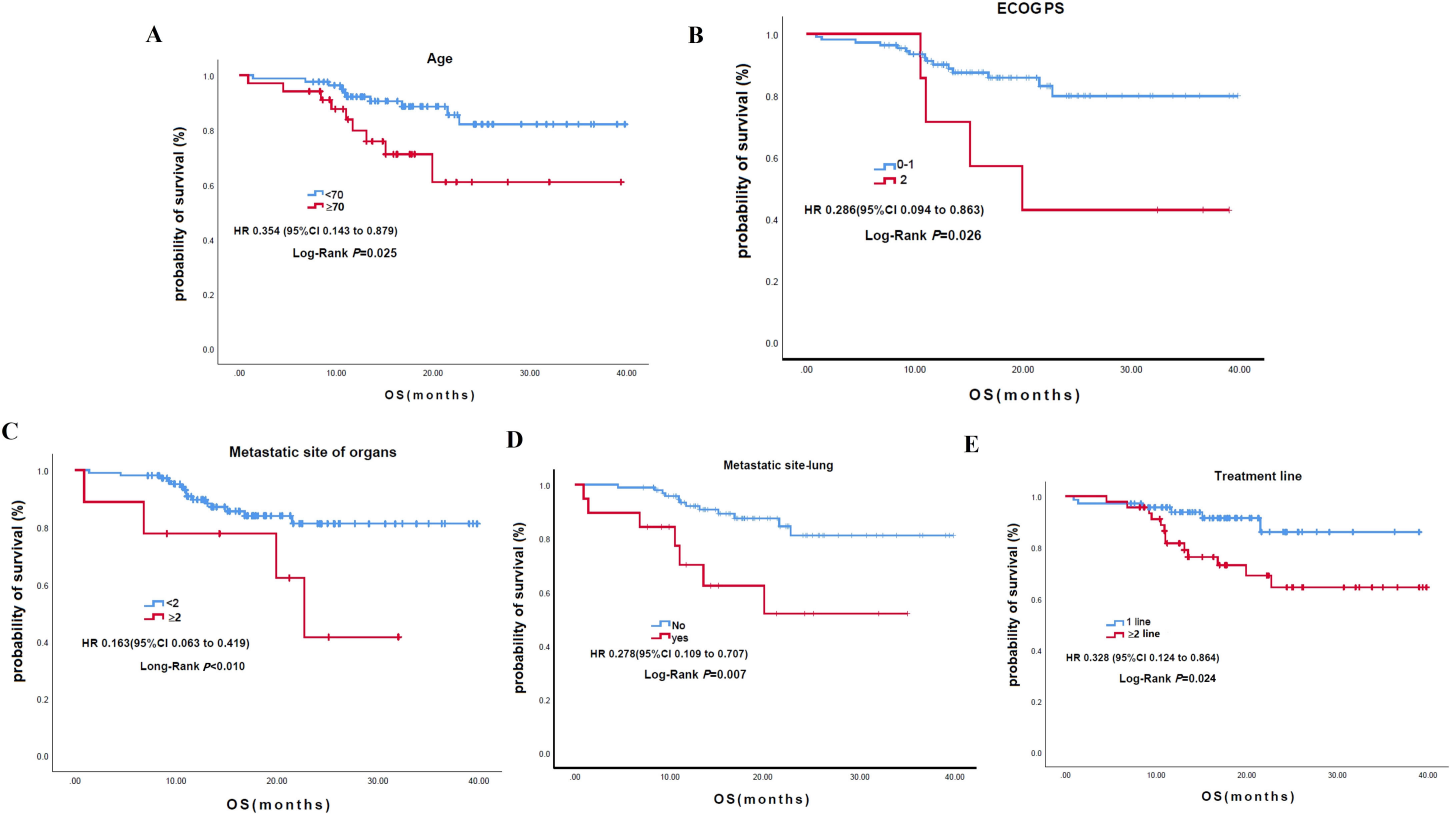
Kaplan-Meier curves for overall survival stratified by clinical characteristics. **(A)** Age. **(B)** ECOG. **(C)** Number of metastatic organs. **(D)** Presence of lung metastases.

The mean OS was not reached (**Figure 2B**). For OS, univariate analysis revealed significantly better OS in younger patients (<70) (HR: 0.354; P = 0.025), those with better ECOG PS 0/1 (HR: 0.286; P = 0.026), fewer than 2 metastatic organs (HR: 0.163; P<0.010), absence of lung metastases (HR: 0.278; P = 0.007), absence of liver metastases (HR: 0.327; P = 0.032), and first-line treatment (HR: 0.328; P = 0.024) (**Figure 4**; **Table 3**). In the multivariable analysis, younger age (<70) (HR: 0.296; P = 0.015), better ECOG PS 0/1 (HR: 0.255; P = 0.022), and absence of lung metastases (HR: 0.228; P = 0.023) remained significant independent predictors of superior OS. Notably, chemotherapy inclusion was associated with significantly worse OS (HR: 4.712; P = 0.039) in the multivariable model (**Table 3**). Other factors were not significant (**Table 3**).

**Table 3:** Univariate and multivariate analysis of OS in the immunotherapy plus chemotherapy subgroup.

Variables	Category	Univariable analysis			*P*	Multivariate analysis			*P*
		HR	95% CI			HR	95% CI		
Age (years)	<70 vs. ≥ 70	0.354	0.143	0.879	0.025	0.296	0.111	0.792	0.015
Sex	Female vs. male	0.913	0.265	3.142	0.885				
Smoking	No vs. yes	0.783	0.297	2.062	0.620				
ECOG PS	0/1 vs. 2	0.286	0.094	0.863	0.026	0.255	0.079	0.824	0.022
Comorbidities	No vs. yes	1.036	0.637	1.682	0.888				
M stage	M0 vs. M1	0.511	0.208	1.260	0.145				
Number of metastatic organs	<2 vs. ≥2	0.163	0.063	0.419	<0.010	2.555	0.443	14.757	0.294
Lung metastasis	No vs. yes	0.278	0.109	0.707	0.007	0.228	0.063	0.816	0.023
Liver metastasis	No vs. yes	0.327	0.118	0.910	0.032	0.313	0.087	1.135	0.077
Bone metastasis	No vs. yes	1.047	0.241	4.551	0.952				
Distant lymph node metastasis	No vs. yes	0.775	0.225	2.666	0.686				
Current regimen	With vs. without chemotherapy	1.600	0.464	5.518	0.456	4.712	1.079	20.569	0.039
Treatment line	1st line vs. ≥2nd line	0.328	0.124	0.864	0.024	0.537	0.187	1.545	0.249

ECOG PS, Eastern Cooperative Oncology Group Performance Status.

### Safety

TRAEs occurred in 8.6% of patients (10/116), most commonly reactive cutaneous capillary endothelial proliferation (3.4%), hypothyroidism (2.6%), dyspnea (0.9%), constipation (0.9%), and other endocrine events (0.9%) (**Table 4**). Grade ≥3 TRAEs were seen in 3.4% (4/116). No cases of severe pneumonitis, myocarditis, and cytopenias were observed. All AEs were manageable, with no drug-related deaths.

**Table 4:** Treatment-related adverse events.

Treatment-related adverse events	Any grade (n, %)	Grade ≥ 3 (n, %)
Pneumonitis	0	0
Dyspnea	1 (0.86)	1 (0.86)
Cough	0	0
Hemoptysis	0	0
Arrhythmias	0	0
Chest discomfort	0	0
Myocarditis	0	0
Elevated liver enzymes	0	0
Hypothyroidism	3 (2.28)	0
Hyperthyroidism	0	0
Anemia	0	0
Leukopenia	0	0
Neutropenia	0	0
Thrombocytopenia	0	0
Fatigue	0	0
Edema	0	0
Anorexia	0	0
Hypoalbuminemia	0	0
Nausea	0	0
Vomiting	0	0
Diarrhea	0	0
Constipation	1 (0.86)	0
Electrolyte imbalance	0	0
Sore throat	0	0
Elevated lactate dehydrogenase	0	0
Rash	0	0
Reactive cutaneous capillary endothelial proliferation	4 (3.44)	3 (2.28)
Myelitis	0	0
Peripheral sensory neuropathy	0	0
Endocrine disorder	1 (0.86)	0

## Discussion

PD-1 inhibitors are promising therapy for ESCC, as they block the PD-1/PD-L1 pathway and restore antitumor immunity. Unlike traditional chemotherapy, which offers limited benefits in advanced ESCC, PD-1 inhibitors—with or without chemotherapy—have shown improved outcomes in phase 3 trials and are now recommended in international guidelines (9–11). This approach addresses the urgent need for better treatment options given the poor prognosis of advanced ESCC (15, 16).

The study reported an ORR of 40.5% in a real-world cohort, which is noteworthy. This ORR is higher than that reported for pembrolizumab monotherapy in the KEYNOTE-181 trial (22% in PD-L1 CPS ≥10 East Asian patients) or nivolumab monotherapy in ATTRACTION-3 (19.3%) for previously treated ESCC (17–19). However, our cohort predominantly received combination therapy (64.7% with chemotherapy), and the ORR is more comparable to, albeit still numerically favorable than, some first-line combination trial arms, such as pembrolizumab plus chemotherapy in KEYNOTE-590 (ORR 45.0%) (9) or nivolumab plus chemotherapy in CheckMate-648 (ORR 47%) (10). The higher ORR observed in our study compared to monotherapy trials may be attributed to the synergistic effects of combination therapy and potentially differences in patient populations, though the lack of PD-L1 expression data in our cohort limits direct comparison with biomarker-stratified trial subgroups.

The PFS was 13.6 months, and the median OS was not reached in our cohort. These results are highly encouraging when compared to historical data from chemotherapy-alone regimens, which typically show a median OS of less than one year (1, 2). Furthermore, the median PFS observed in our study appears favorable when compared to first-line PD-1 inhibitor plus chemotherapy arms in pivotal trials, e.g., KEYNOTE-590 (median PFS 6.3 months) (9), CheckMate-648 (median PFS 5.8 months) (8), and RATIONALE-306 (median PFS 7.3 months) (11). Similarly, the median OS (not reached) appears promising compared to these trials (median OS around 13–17 months for PD-1 combo arms). Several factors might contribute to these favorable survival outcomes in our real-world cohort, including patient selection (e.g., a high proportion of ECOG PS 1 patients), specific combination strategies employed, regional practice variations, or potentially longer follow-up for some surviving patients influencing the mean OS. However, direct cross-study comparisons should be made with caution due to inherent differences in study design and patient populations.

The study included patients receiving PD-1 inhibitors as both first-line and subsequent-line treatments. Previous research has consistently shown that PD-1 inhibitors are more effective when administered in earlier treatment lines. Our study also demonstrated that first-line treatment was associated with better PFS and OS (though not multivariate for OS) compared to treatment in later lines. This suggests that the timing of immunotherapy initiation may significantly moderate treatment outcomes, with earlier intervention being more effective. The rationale behind this observation may be related to the fact that in earlier stages of the disease, the tumor burden is relatively lower, and the immune system may be less compromised, thus allowing for a more robust antitumor immune response to be elicited by PD-1 inhibitors. The combination of PD-1 inhibitors with chemotherapy has been established as the new global standard for first-line systemic therapy in advanced ESCC (9–11, 20) (21).

The study’s diverse patient population mirrors real-world clinical practice, highlighting the importance of considering age, performance status, and comorbidities when selecting patients for PD-1 inhibitor therapy. Younger age (<70), better ECOG (0/1), and fewer metastatic organs were significant predictors of improved survival, suggesting that patient-specific factors influence treatment outcomes. These findings support personalized treatment strategies and refined immunotherapy selection criteria. However, chemotherapy combined with immunotherapy seemed linked to worse OS (P = 0.039), possibly due to confounding factors or selection bias. This result, contrary to clinical trial data, requires further investigation in larger, more detailed studies.

Our real-world findings support the immune checkpoint hypothesis by showing that PD-1 inhibitors yield strong ORR and DCR even in more heterogeneous patient populations, including those with advanced disease or comorbidities. Notably, patients with lung or bone metastases had poorer outcomes, aligning with the tumor microenvironment model that suggests immune-suppressive elements in metastatic sites can limit immunotherapy efficacy. This highlights the need for tailored approaches to overcome these barriers in certain metastatic settings.

Regarding treatment modalities, no significant PFS or OS differences were observed between monotherapy and combination therapy, likely due to small monotherapy sample size (only 15) and selection bias. The landmark PFS/OS rates (12/24 months: 60.4%/31.0%; 88.7%/76.3%) in our study were higher than those reported in KEYNOTE-590 (9), reflecting favorable baseline status and proactive AE management.

TRAEs occurred in 8.6% of patients, with grade ≥3 events in 3.4%. This is lower than rates reported in major clinical trials, where severe TRAEs often reach 40–60% with PD-1 inhibitor combinations (9, 10), and 18% with nivolumab monotherapy (18). This discrepancy is primarily due to the retrospective design. Mild adverse events may not have been systematically documented, and this study lacked the prospective, standardized monitoring protocols used in clinical trials. Other factors may include patient selection, treatment regimens, or effective AE management. Importantly, no treatment-related deaths were seen, supporting a favorable and manageable safety profile for clinical practice.

This real-world study of PD-1 inhibitors in ESCC patients offers valuable outcome and safety data from a single Chinese center, but several limitations affect validity. The single-center retrospective design introduced potential selection bias and limited the generalizability of the results. Additionally, the study relied on EMR data without key biomarker data (notably lacking PD-L1, tumor mutational burden [TMB], microsatellite instability [MSI] status), which represented a particularly important limitation given PD-L1’s role as a predictive biomarker in ESCC. The absence of these biomarker data limits our ability to identify patients most likely to benefit from PD-1 inhibitor therapy. Other limitations include potential confounding factors, short follow-up for some patients, and the use of different PD-1 inhibitors (camrelizumab, sintilimab tislelizumab) with various combination therapies, which could influence observed efficacy and safety. The absence of a control group (e.g., patients treated with chemotherapy or surgery alone) further limits our ability to assess the relative efficacy of PD-1 inhibitors. Future multi-center, prospective studies with appropriate control groups and comprehensive biomarker assessments (including PD-L1 expression, TMB, and MSI status) are needed to validate these findings, refine patient selection strategies, and provide more robust evidence for clinical practice.

## Conclusions

This retrospective study found that PD-1 inhibitor-based therapy in real-world ESCC patients led to an ORR of 40.5%, median PFS of 13.6 months, and a low rate of severe TRAEs (3.4%). Improved survival was seen in younger patients, those with better performance status, fewer metastases, and first-line treatment. These results suggest PD-1 inhibitors can benefit selected ESCC patients, but larger prospective studies are needed to confirm these findings and identify predictive biomarkers for personalized therapy.

## Data Availability

The original contributions presented in the study are included in the article/**Supplementary Material**. Further inquiries can be directed to the corresponding author.
