# Supplementary figures and images for: Real-world efficacy and safety of PD-1 inhibitors in patients with advanced esophageal squamous cell carcinoma: a single-center retrospective analysis
Source: Front Oncol. 2025 Oct 29;15:1658010. doi: 10.3389/fonc.2025.1658010 (PMC12605918; doi:10.3389/fonc.2025.1658010)

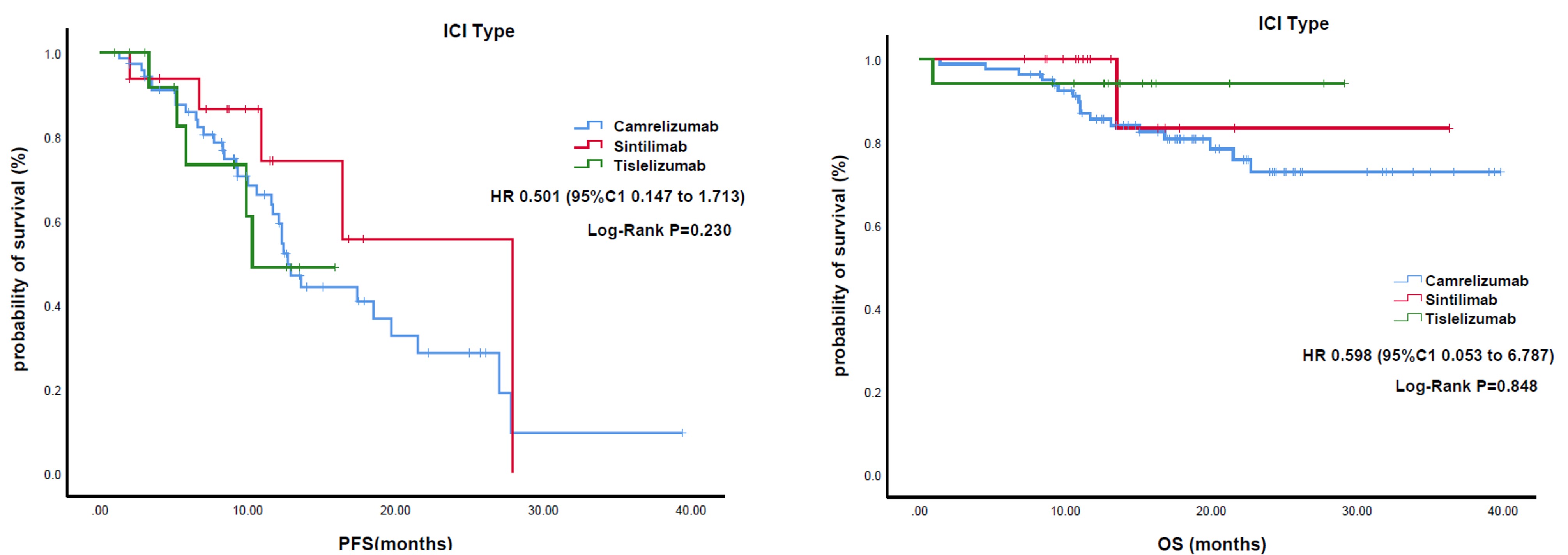

Supplement: Supplementary file 1 [file Image1.jpeg]

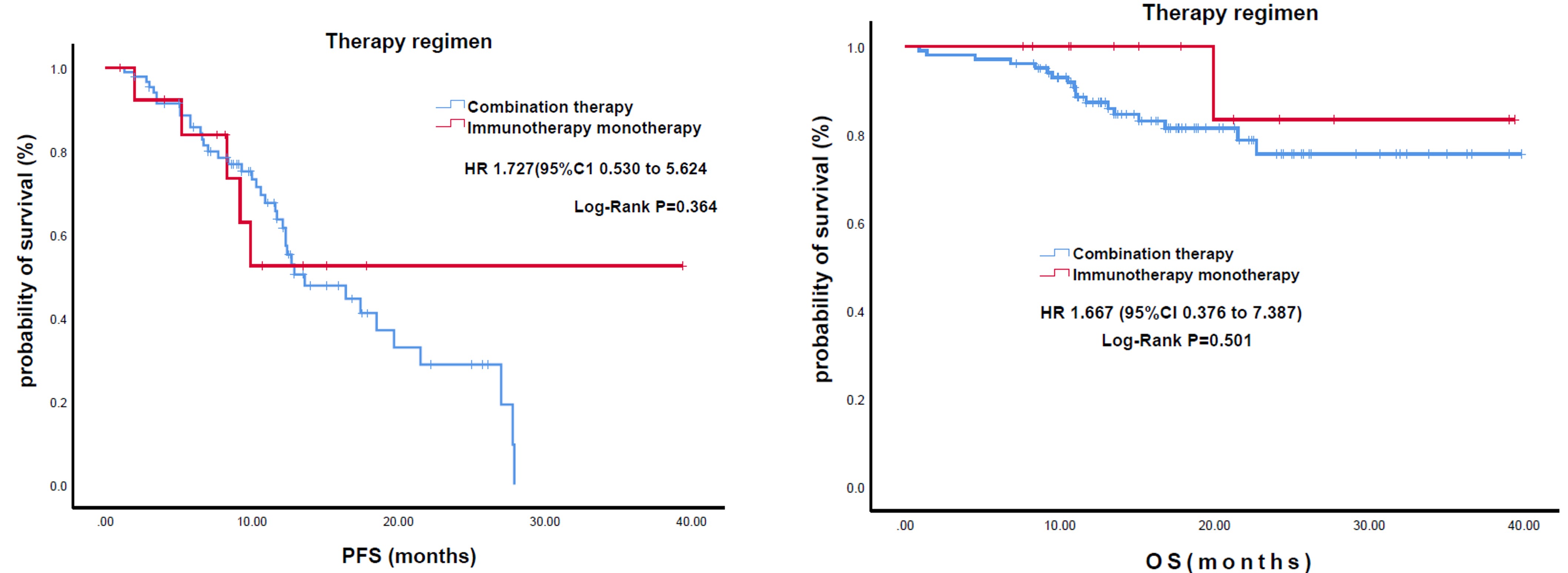

Supplement: Supplementary file 2 [file Image2.jpeg]
